# Myosin-X is dispensable for spindle morphogenesis and positioning in the mouse oocyte

**DOI:** 10.1242/dev.199364

**Published:** 2021-04-15

**Authors:** Flora Crozet, Christelle Da Silva, Marie-Hélène Verlhac, Marie-Emilie Terret

**Affiliations:** CIRB, Collège de France, UMR7241/U1050, 75005 Paris, France

**Keywords:** Myosin-X, Mouse oocyte, Spindle positioning, F-actin

## Abstract

Off-center spindle positioning in mammalian oocytes enables asymmetric divisions in size, which are important for subsequent embryogenesis. The migration of the meiosis I spindle from the oocyte center to its cortex is mediated by F-actin. Specifically, an F-actin cage surrounds the microtubule spindle and applies forces to it. To better understand how F-actin transmits forces to the spindle, we studied a potential direct link between F-actin and microtubules. For this, we tested the implication of myosin-X, a known F-actin and microtubule binder involved in spindle morphogenesis and/or positioning in somatic cells, amphibian oocytes and embryos. Using a mouse strain conditionally invalidated for myosin-X in oocytes and by live-cell imaging, we show that myosin-X is not localized on the spindle, and is dispensable for spindle and F-actin assembly. It is not required for force transmission as spindle migration and chromosome alignment occur normally. More broadly, myosin-X is dispensable for oocyte developmental potential and female fertility. We therefore exclude a role for myosin-X in transmitting F-actin-mediated forces to the spindle, opening new perspectives regarding this mechanism in mouse oocytes, which differ from most mitotic cells.

## Abstract (precis)

**Summary:** Cortical spindle positioning in mammalian oocytes relies on the interplay between actin and the microtubule spindle. Myosin-X, an obvious candidate for linking these two cytoskeletal elements, is dispensable in mouse oocytes.

## INTRODUCTION

Spindle positioning is key for the development of eukaryotic organisms as it defines the geometry of cell division. It can trigger either redundancy or divergence of fate between daughter cells, following a symmetric versus an asymmetric division (for reviews, see Morin and Bellaïche, 2011; Di Pietro et al., 2016). Owing to their two successive asymmetric divisions in size (meiosis I and II), mammalian oocytes are relevant models for studying spindle off-centering mechanisms. In meiosis I, it is a process that has not yet been fully deciphered, consisting of the migration of the spindle from the cell center to the cortex (Verlhac et al., 2000). The asymmetric divisions of the oocyte provide the prospective mother with one big egg and two tiny polar bodies, a discrepancy in size that is important for keeping the largest proportion of maternal stores needed for embryogenesis in the fertilizable egg (Kyogoku and Kitajima, 2017).

As with most mitotic cells (Morin and Bellaïche, 2011; Di Pietro et al., 2016), spindle positioning in mammalian oocytes relies on cortical inputs and forces applied to the spindle, and hence depends on the cell mechanical properties. Pioneering studies in murine oocytes have highlighted the importance of tight regulation of cortical mechanics to properly transmit forces to the spindle, enabling an asymmetric division in size and a good oocyte quality (Chaigne et al., 2013, 2015; Bennabi et al., 2020). Cortical mechanics is a very good predictor of oocyte developmental potential in humans and mice (Yanez et al., 2016). Strikingly, a weakened transmission of forces in extra-soft engineered oocytes prevents spindle off-centering (Chaigne et al., 2015) and induces chromosome misalignment, causing aneuploidy (Bennabi et al., 2020). The forces applied to mouse oocyte spindles are mediated by filamentous actin (F-actin) bypassing the absence of canonical centrosomes and the consequent lack of astral microtubules (Szollosi et al., 1972; Longo and Chen, 1985), which are the main effectors of spindle positioning in mitotic cells (Théry et al., 2005; Toyoshima and Nishida, 2007; Fink et al., 2011; Morin and Bellaïche, 2011; Woolner and Papalopulu, 2012; Kwon et al., 2015; Di Pietro et al., 2016). A dynamic cytoplasmic F-actin network, including an actin-cage surrounding the spindle (Azoury et al., 2008; Schuh and Ellenberg, 2008; Chaigne et al., 2013, 2015; Mogessie and Schuh, 2017), is polymerized by the straight actin nucleators formin 2 (Leader et al., 2002; Dumont et al., 2007a; Azoury et al., 2008; Schuh and Ellenberg, 2008), spire 1 and spire 2 (Pfender et al., 2011). It anchors the spindle to the cortex and off-centers it through myosin II-generated pulling forces at spindle poles (Schuh and Ellenberg, 2008; Chaigne et al., 2013; Bennabi et al., 2020). A subcortical Arp2/3-dependent F-actin network (Chaigne et al., 2013, 2015), progressively nucleated after nuclear envelope breakdown (NEBD), remodels cortical properties by chasing myosin II from the cortex, resulting in lower cortical tension (Larson et al., 2010; Chaigne et al., 2013, 2015). Working in synergy with the cytoplasmic F-actin, the subcortical F-actin network increases an imbalance of pulling forces at the spindle poles (Chaigne et al., 2015). This imbalance first originates from the slightly off-center assembly of the spindle before the onset of migration (Verlhac et al., 2000) and its initial pushing (Li et al., 2008; Yi et al., 2013; Duan et al., 2020), resulting in one pole closer to the cortex (the leading pole). Following cortex softening, this imbalance of forces is further amplified by the cytoplasmic F-actin-mediated stretching of the subcortical F-actin towards the leading spindle pole, likely providing spindle-localized myosin II with greater substrate and accelerating spindle migration (Chaigne et al., 2015).

Although the cytoplasmic F-actin involvement in coupling cortical mechanics to spindle off-centering is well established in mouse oocytes, how it directly transmits forces to the spindle remains elusive. Intriguingly, the actin cage perfectly surrounds the microtubule spindle and numerous actin filaments penetrate it (Azoury et al., 2008; Schuh and Ellenberg, 2008; Mogessie and Schuh, 2017), suggesting a possible inter-cytoskeleton crosstalk at the spindle. Moreover, artificial F-actin enrichment at the meiotic spindles in murine eggs increases the bundling of microtubules and affects their dynamics (Mogessie and Schuh, 2017), supporting the ability of F-actin to transmit mechanical constrains to the microtubules and suggesting a spindle-localized F-actin/microtubule crosslinker in mouse oocytes. In this study, we addressed the role of a known F-actin and microtubule binder, the unconventional myosin-X (MYO10), in this process. MYO10 is a MyTH4-FERM myosin (myosin tail homology 4 - band 4.1, ezrin, radaxin, moesin) (Berg et al., 2000; Hirano et al., 2011) capable of binding both to F-actin via its head motor domain (Homma et al., 2001) and to microtubules via its MyTH4-FERM domain (Weber et al., 2004; Hirano et al., 2011). Numerous studies have highlighted MYO10 involvement in spindle dynamics, orientation and positioning in mitotic cells (Toyoshima and Nishida, 2007; Woolner et al., 2008; Liu et al., 2012; Woolner and Papalopulu, 2012; Kwon et al., 2015; Sandquist et al., 2016, 2018). It has also been involved in nuclear anchoring and spindle rotation in *Xenopus* oocytes (Weber et al., 2004), as well as in spindle morphogenesis and integrity in *Xenopus* oocytes and embryos (Weber et al., 2004; Woolner et al., 2008; Sandquist et al., 2016). We therefore investigated whether MYO10 directly links F-actin to spindle microtubules in mouse oocytes and thus participates in spindle off-centering. We took advantage of the *Cre*-*loxP* system to generate a mouse strain conditionally invalidated for MYO10 in oocytes and assessed spindle morphogenesis and positioning in oocytes lacking MYO10, as well as their subsequent developmental capacity. Our results show that MYO10 is present in mouse oocytes but not enriched at the spindle in meiosis I. Importantly, MYO10 is dispensable for actin organization, spindle morphogenesis and positioning in oocytes, and more generally not essential for female fertility.

## RESULTS

### MYO10 does not localize to the meiosis I spindles

**Fig. 1.**
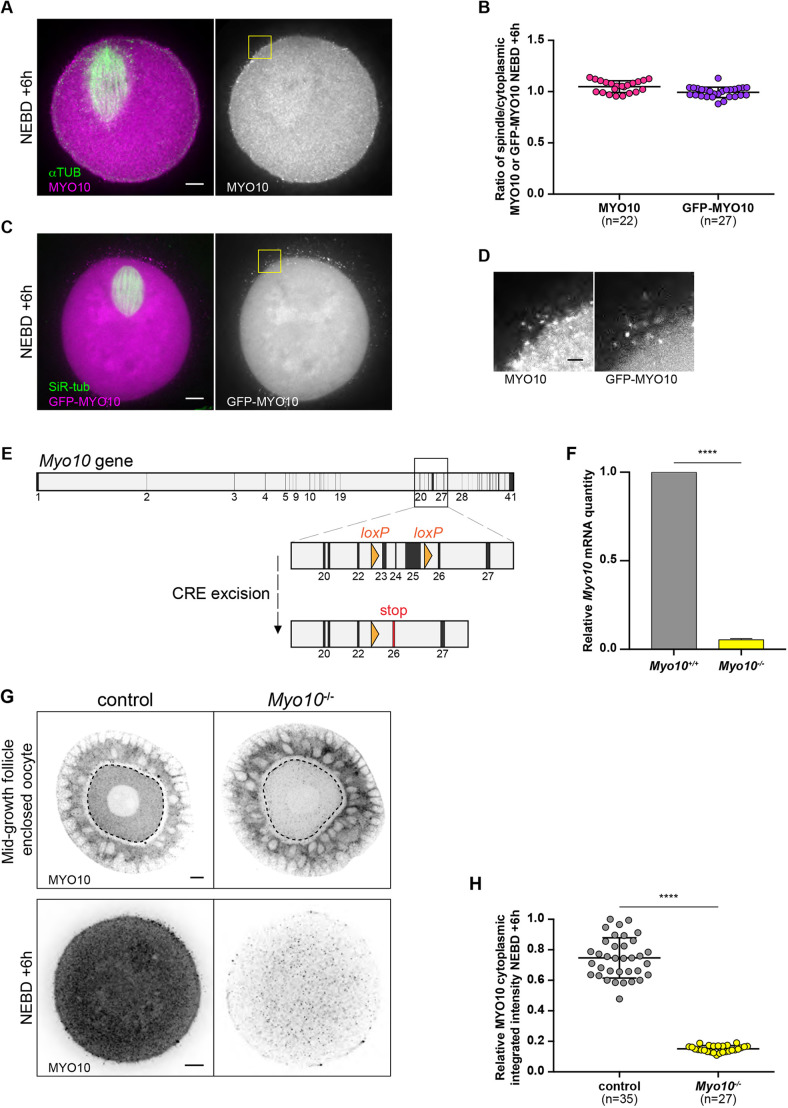
**MYO10 does not accumulate at the meiosis I spindles.** (A) Confocal spinning disk images of an oocyte fixed at NEBD+6 h stained for α-tubulin (αTUB) and myosin-X (MYO10). Left, merge of αTUB (green) and MYO10 (magenta) staining. Right, MYO10 staining only. Scale bar: 10 µm. (B) Scatter plot representing the ratio of spindle and cytoplasmic MYO10 fluorescence intensities at NEBD+6 h in fixed oocytes labeled with MYO10 and αTUB (pink), and in live oocytes expressing GFP-MYO10 and incubated with SiR-tubulin (purple). *n* is the number of oocytes analyzed. Data are from three (fixed oocytes) and two (live oocytes) independent experiments. Data are mean and s.d. with individual data points plotted. (C) Confocal spinning disk images of an oocyte at NEBD+6 h microinjected with GFP-MYO10 and incubated with SiR-tubulin (SiR-tub) to label the microtubules. Left, merge of SiR-tub (green) and GFP-MYO10 (magenta) staining. Right, GFP-MYO10 staining only. Scale bar: 10 µm. (D) Confocal spinning disk images showing cortical/membrane foci of endogenous (left) and exogenous (right) MYO10. Images corresponding to the areas outlined with yellow squares in A and C. Scale bar: 2 µm. (E) *Cre*-*loxP*-mediated depletion of MYO10 in oocytes. The *Myo10* gene with exons in black and introns in gray based on the Ensembl database. *loxP* sites, shown as orange triangles, flank exons 23 to 25. *Zp3*-mediated CRE expression in oocytes allows *Myo10* excision at the *loxP* sites. The depletion of exons 23-25 leads to a frame-shift and the appearance of a stop codon at exon 26. (F) Bar chart representing *Myo10* mRNA levels in *Myo10^−/−^* oocytes (from *Myo10^flox/flox^; Cre^+^* female mice, yellow) relative to *Myo10^+/+^* oocytes (from *Myo10^flox/flox^; Cre^−^* female mice, gray). *Myo10^+/+^* normalized mean=1. *Myo10^−/−^* normalized mean=0.05±0.005 (normalized s.e.m.). RT-qPCRs were performed on two biological replicates using 30 oocytes for each condition from two females; two technical replicates were carried out. The statistical significance of the differences was assessed with a two-tailed unpaired *t*-test, ****P<0.0001. Error bar for the *Myo10^−/−^* group represents s.e.m. (G) Confocal spinning disk images of fixed oocytes at mid-growth surrounded by granulosa cells (granulosa-oocyte complexes, upper images) and fully grown oocytes fixed at NEBD+6 h (lower images) stained for MYO10. Controls are on the left and *Myo10^−/−^* oocytes on the right. The black dotted lines delimit the oocytes in the complexes. The images show the equatorial plane of the oocytes. Scale bars: 10 µm. (H) Scatter plots of the relative integrated intensities of cytoplasmic MYO10 labeling observed at NEBD+6 h in control (gray) and *Myo10^−/−^* (yellow) oocytes. *n* is the number of oocytes analyzed. Data are from three independent experiments. Data are mean±s.d. with individual data points plotted. The statistical significance of the differences was assessed with a two-tailed unpaired *t*-test with Welch's correction, ****P<0.0001.

If MYO10 crosslinks F-actin to microtubules in mouse oocytes, it might be enriched at the spindle. In several models, MYO10 was detected at both mitotic and meiotic spindles (Weber et al., 2004; Woolner et al., 2008; Woolner and Papalopulu, 2012; Sandquist et al., 2016, 2018; Brieño-Enríquez et al., 2017; So et al., 2019), and more specifically often accumulated at spindle pole regions in the vicinity of cortical/spindle F-actin (Weber et al., 2004; Woolner et al., 2008). Contrary to what was previously described in mouse oocytes (Brieño-Enríquez et al., 2017; So et al., 2019), we did not detect by immunofluorescence any endogenous MYO10 accumulation at the meiosis I spindles (Fig. 1A). We quantified MYO10 spindle enrichment by measuring the ratio of the spindle and cytoplasmic MYO10 fluorescence intensities. This ratio was close to 1 (Fig. 1B left, 1.05±0.06), showing that endogenous MYO10 is not enriched at the spindle. In addition, no specific labeling of MYO10 in the cytoplasm was observed, with a signal that was mostly diffuse. In previous articles (Brieño-Enríquez et al., 2017; So et al., 2019), the authors used different antibodies and immunostaining protocols than ours, which could explain the different localizations observed between their studies and ours. However, these studies were not intended to address the role of myosin-X in oocytes, so the specificity of their staining was not tested on oocytes depleted for myosin-X (genetically or using siRNA) as we do here. Indeed, our MYO10 antibody was further validated in our mouse strain conditionally invalidated for MYO10 in oocytes (*Myo10^−/−^*, see Materials and Methods and Fig. 1E-H). We observed a decrease in the intensity of MYO10 labeling in both mid-grown *Myo10^−/−^* oocytes enclosed in an environment of otherwise wild-type granulosa cells and fully grown *Myo10^−/−^* oocytes compared with their respective controls (Fig. 1G). This intensity was fivefold lower in the cytoplasm of *Myo10^−/−^* oocytes than in controls at NEBD+6 h (Fig. 1G, lower images and quantification in H; control, 0.75±0.13; *Myo10^−/−^*, 0.15±0.02). Conversely, we detected MYO10 in the wild-type granulosa cells surrounding *Myo10^−/−^* oocytes at mid-growth, as in those surrounding control oocytes of the same stage (Fig. 1G, upper images). This further confirms the specificity of the MYO10 antibody and also shows the efficiency of our genetic approach that allowed the specific depletion of MYO10 in oocytes from the mid-growth stage of their development. In parallel, we microinjected exogenous MYO10 tagged with GFP (GFP-MYO10) into prophase I oocytes and monitored its localization throughout meiosis I. Similar to endogenous MYO10, GFP-MYO10 was not enriched at the meiosis I spindles (Fig. 1B, right, C and Movie 1; ratio of spindle and cytoplasmic GFP-MYO10 fluorescence intensities of 0.99±0.05) or at any other cytoplasmic location. Supporting the robustness of our MYO10 detection tools, we observed that both endogenous and exogenous MYO10 (Fig. 1D, left and right, respectively) accumulated in foci in the vicinity of the oocyte cortex, a pattern of localization consistent with previous studies in somatic cells (Berg et al., 2000; Liu et al., 2012; Kwon et al., 2015). To conclude, and in contrast to other models, MYO10 is present in mouse oocytes but is not enriched at the spindle.

### Spindle morphogenesis is normal in MYO10 depleted oocytes

**Fig. 2.**
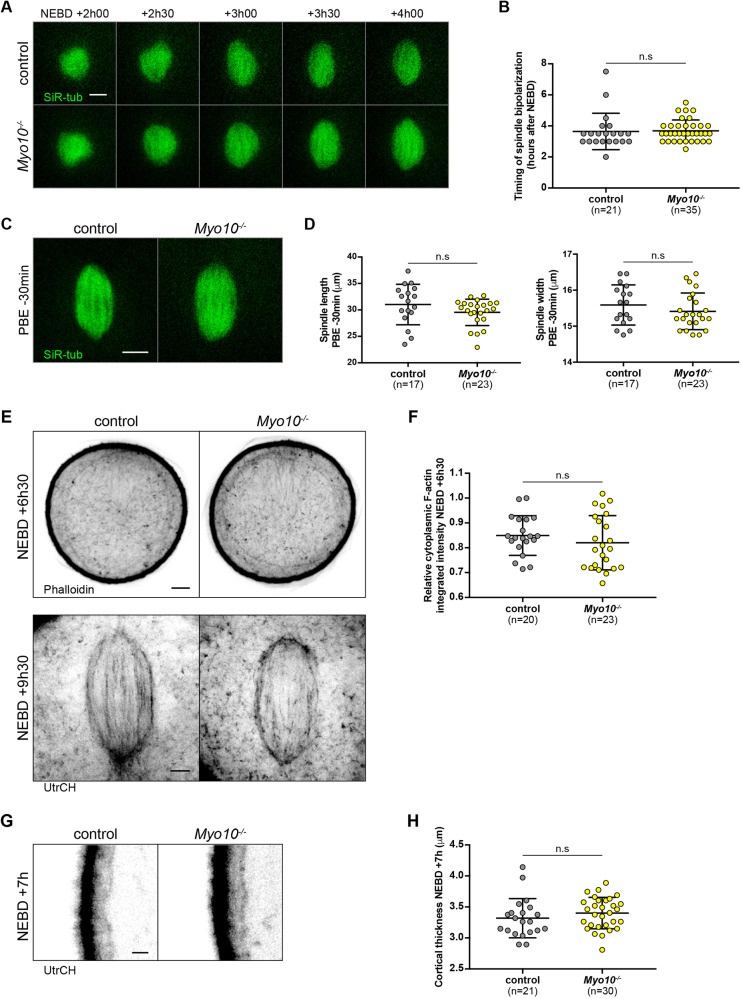
**Meiosis I spindles and F-actin networks are properly formed in *Myo10^−/−^* oocytes.** (A) Stills from time-lapse spinning disk movies showing microtubules labeled with SiR-tub (green) forming a ball that progressively bipolarizes to form the meiosis I spindle in a control (upper images) and a *Myo10^−/−^* (lower images) oocyte. Scale bar: 10 µm. (B) Scatter plots of the timings of spindle bipolarization (hours after NEBD) in control (gray) and *Myo10^−/−^* (yellow) oocytes. *n* is the number of oocytes analyzed. Data are from three independent experiments (mean±s.d. with individual data points plotted). The statistical significance of the differences was assessed with a two-tailed Mann-Whitney test, *P*=0.2948. (C) Confocal spinning disk images of a control (left) and a *Myo10^−/−^* (right) spindle 30 min before polar body extrusion (PBE) labeled with SiR-tub (green). Scale bar: 10 µm. (D) Scatter plots of spindle length (left chart) and width (right chart) 30 min before PBE in control (gray) and *Myo10^−/−^* (yellow) oocytes. *n* is the number of oocytes analyzed. Data are from five independent experiments (mean±s.d. with individual data points plotted). The statistical significance of the differences was assessed with a two-tailed Mann-Whitney test, *P*=0.1053 for the length and with a two-tailed unpaired *t*-test, *P*=0.2995 for the width. (E) The upper images are oocytes fixed at NEBD+6 h30 showing cytoplasmic F-actin meshes labeled with phalloidin. The images correspond to the equatorial plane of the oocytes. Left, control; right, *Myo10^−/−^*. Scale bar: 10 µm. The lower images are confocal spinning disk images of oocytes at NEBD+9 h30 microinjected with GFP-UtrCH (UtrCH) showing actin cages in control (left) and *Myo10^−/−^* (right) oocytes. Scale bar: 5 µm. (F) Scatter plots of the relative integrated cytoplasmic intensity from the labeling with phalloidin at NEBD+6 h30 in control (gray) and *Myo10^−/^*^−^ (yellow) oocytes. *n* is the number of oocytes analyzed. Data are from four independent experiments (mean±s.d. with individual data points plotted). The statistical significance of the differences was assessed with a two-tailed unpaired *t*-test, *P*=0.3315. (G) Confocal spinning disk images focused on the equatorial plane cortex of a control (left) and a *Myo10^−/−^* (right) oocyte microinjected with UtrCH at NEBD+7 h. Scale bar: 2 µm. (H) Scatter plots of the measure of cortex thickness at NEBD+7 h in control (gray) and *Myo10^−/^*^−^ (yellow) oocytes. *n* is the number of oocytes analyzed. Data are from two independent experiments (mean±s.d. with individual data points plotted). The statistical significance of the differences was assessed with a two-tailed Mann-Whitney test, *P*=0.1322.

MYO10 plays a role in spindle morphogenesis and integrity in various models. MYO10 disruption, using morpholino oligonucleotide-based approaches, dominant-negative constructs or neutralizing antibodies, alters spindle assembly in *Xenopus* oocytes (Weber et al., 2004), and triggers both spindle lengthening (Woolner et al., 2008; Woolner and Papalopulu, 2012) and pole fragmentation (Woolner et al., 2008; Sandquist et al., 2016) in *Xenopus* embryos. In somatic cells, however, spindle length (Toyoshima and Nishida, 2007; Liu et al., 2012) and pole integrity (Kwon et al., 2015) are normal when MYO10 is knocked down. In murine oocytes, spindle morphogenesis starts with the formation of a microtubule ball assembled around the chromosomes following NEBD that progressively bipolarizes (Schuh and Ellenberg, 2007; Dumont et al., 2007b; Kitajima et al., 2011; Bennabi et al., 2016). We followed spindle morphogenesis using live spinning disk microscopy during meiosis I in control and *Myo10*^−/−^ oocytes incubated with SiR-tubulin to label the microtubules. Both the bipolarization timing of meiosis I spindles (Fig. 2A,B; control 3.64±1.17 h, *Myo10^−/−^* 3.69±0.70 h; Movie 2), their inter-polar length and width 30 min before polar body extrusion (Fig. 2C,D; control, 31.02±3.82 µm; *Myo10^−/−^*, 29.54±2.49 µm for spindle length; control, 15.59±0.56 µm; *Myo10^−/−^* 15.41±0.51 µm for spindle width) were not significantly different between control and *Myo10^−/−^* oocytes. MYO10 therefore does not play a role in spindle morphogenesis and integrity in mouse oocytes.

### F-actin is properly organized in MYO10-depleted oocytes

Owing to the role of MYO10 in actin organization in *Xenopus* oocytes and somatic cells (Weber et al., 2004; Toyoshima and Nishida, 2007), we also assessed the integrity of the cytoplasmic F-actin in *Myo10*^−/−^ oocytes by immunofluorescence in fixed cells using phalloidin staining (Fig. 2E, upper images) and in live oocytes microinjected with the F-actin probe GFP-UtrCH (Fig. 2E, lower images). The cytoplasmic F-actin meshes were not altered in meiosis I *Myo10^−/−^* oocytes, as evidenced by a cytoplasmic F-actin intensity not significantly different from controls (Fig. 2E, upper images, F; control, 0.85±0.08; *Myo10^−/−^*, 0.82±0.11). The actin cages enclosing the meiosis I spindles were also properly shaped in *Myo10^−/−^* oocytes (Fig. 2E, lower images), arguing that MYO10 does not play a role in assembling such structures. In addition, we assessed the integrity of the subcortical F-actin network, which mediates spindle positioning through cortex softening, in GFP-UtrCH-injected *Myo10^−/−^* oocytes. No defects in the subcortical F-actin network were observed in such a MYO10 depletion context, as indicated by a thickness comparable with controls at NEBD+7 h in *Myo10^−/−^* oocytes (Fig. 2G,H; control, 3.32±0.32 µm; *Myo10^−/−^*, 3.40±0.25 µm). Interestingly, the lack of impact of MYO10 depletion on F-actin networks is consistent with earlier studies and is not specific to mouse oocytes. Subcortical F-actin is not affected by MYO10 knockdown in terms of assembly and cellular localization in somatic cells (Kwon et al., 2015), nor are the F-actin cables surrounding the spindle in *Xenopus* embryos (Woolner et al., 2008).

### The spindle migrates in MYO10 depleted oocytes

**Fig. 3.**
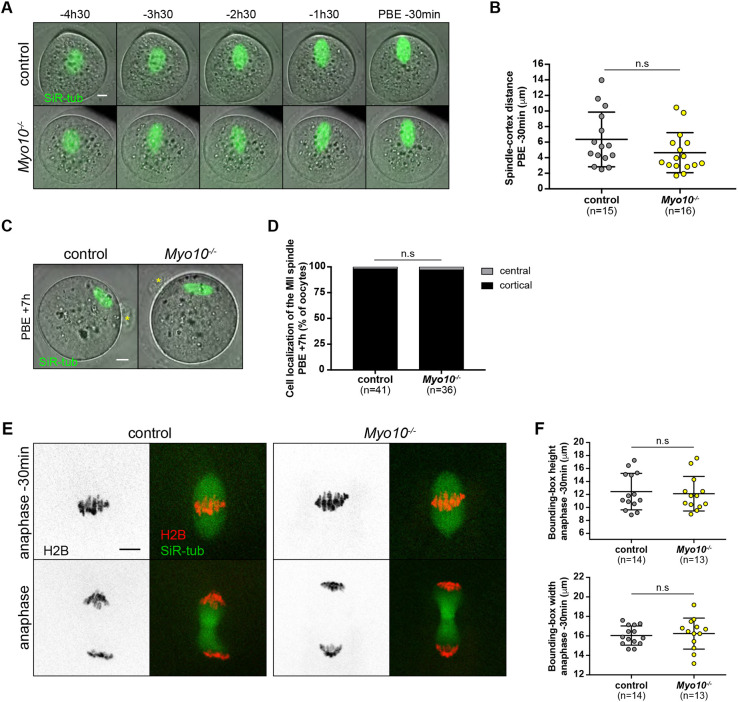
**Force transmission to the spindle and chromosomes is unaffected by MYO10 depletion.** (A) Stills from time-lapse spinning disk movies showing meiosis I spindles labeled with SiR-tub (green) merged to their corresponding bright-field images. Control oocyte in upper images, *Myo10^−/−^* in lower images. Scale bar: 10 µm. (B) Scatter plots of the distance between the leading spindle pole and the cortex 30 min before polar body extrusion (PBE) in control (gray) and *Myo10^−/−^* (yellow) oocytes. *n* is the number of oocytes analyzed. Data are from five independent experiment (mean±s.d. with individual data points plotted). The statistical significance of the differences was assessed with a two-tailed unpaired *t*-test, *P*=0.1332. (C) Confocal spinning disk images showing the meiosis II spindles 7 h after the first polar body extrusion (PBE) labeled with SiR-tub (green) and merged to their corresponding bright-field images. The control oocyte is on the left; the *Myo10*^−/−^ oocyte on the right. The yellow asterisks indicate the first polar bodies. Scale bar: 10 µm. (D) Stacked bars of the cell localization of the meiosis II (MII) spindles as a percentage of the oocytes. Gray indicates the meiosis II spindles centrally located in the metaphase II-arrested oocytes; black indicates the meiosis II spindles anchored to the cortex. *n* is the number of oocytes analyzed. The data are from seven independent experiments. The statistical significance of the differences was assessed with a two-sided Fisher's exact test, *P*>0.9999. (E) Stills from time-lapse spinning disk movies showing meiosis I chromosome alignment (upper images, 30 min before anaphase) and segregation (lower images, anaphase) in control (left panel) and *Myo10^−/−^* (right panel) oocytes. Oocytes were microinjected with H2B-RFP (H2B, black or red) and incubated with SiR-tub (green). Scale bar: 10 µm. (F) Scatter plots of the height (upper chart) and the width (lower chart) of the bounding boxes framing the chromosomes 30 min before anaphase in control (gray) and *Myo10^−/−^* (yellow) oocytes. *n* is the number of oocytes analyzed. Data are from three independent experiments (mean±s.d. with individual data points plotted). The statistical significance of the differences was assessed with a two-tailed unpaired *t*-test, *P*=0.7601 for the height and *P*=0.6903 for the width.

In different studies, conducted in a wide variety of models ranging from *Xenopus* oocytes, epithelia to somatic cells, MYO10 was described as being required for proper spindle movement in the cell (Weber et al., 2004; Toyoshima and Nishida, 2007; Woolner et al., 2008; Woolner and Papalopulu, 2012; Liu et al., 2012; Kwon et al., 2015; Sandquist et al., 2016, 2018). Interestingly, MYO10 and F-actin disruption leads to similar defects in spindle orientation in mammalian cells (Toyoshima and Nishida, 2007; Kwon et al., 2015) and aberrant cortical anchoring in *Xenopus* embryos (Woolner et al., 2008), suggesting that they work synergistically in such processes. In mouse oocytes, F-actin disruption impairs spindle migration in meiosis I (Longo and Chen, 1985; Verlhac et al., 2000). We thus analyzed spindle positioning in MYO10-depleted oocytes in order to assess whether MYO10 transmits forces to the spindle. We followed the spindle migration by live spinning disk microscopy during meiosis I in control and *Myo10^−/−^* oocytes incubated with SiR-tubulin to label the microtubules. Unlike the latter models, spindle positioning was unaltered by MYO10 depletion in mouse oocytes (Fig. 3A,B; Movie 3). Spindle migration and cortical positioning, assessed by measuring the distance between the cortex and the leading spindle pole 30 min before polar body extrusion (Fig. 3B; control, 6.35±3.52 µm; *Myo10^−/−^*, 4.65±2.58 µm), were not significantly different between control and *Myo10^−/−^* oocytes. In addition, we examined the cortical position of the spindle in metaphase II-arrested oocytes by live spinning disk microscopy (Fig. 3C). The majority of the arrested control (97.56%) and MYO10 depleted (97.22%) oocytes showed an off-centered meiosis II (MII) spindle 7 h after polar body extrusion (Fig. 3D). These results show that MYO10 does not play a role in the cortical positioning of the meiotic spindles in mouse oocytes.

### Chromosomes are well aligned in MYO10 depleted oocytes

A weakened transmission of forces to the spindle has no effect on spindle morphogenesis but alters meiosis I chromosome alignment in mouse oocytes engineered to become extra soft (Bennabi et al., 2020). We thus assessed chromosome behavior in *Myo10*^−/−^ oocytes, testing whether MYO10 depletion might affect force transmission, if not to the spindle, at least to the chromosomes. We microinjected control and *Myo10^−/−^* oocytes with H2B-RFP to label their chromosomes, incubated them with SiR-tubulin to label the microtubules and followed them by live spinning disk microscopy before polar body extrusion. We observed no defects in the alignment or segregation of meiosis I chromosomes in *Myo10^−/−^* oocytes (Fig. 3E,F; Movie 4). Chromosome alignment was evaluated 30 min before anaphase by framing all chromosomes in a single rectangle (referred to as the bounding box as in Bennabi et al., 2020) and by measuring the bounding box height (Fig. 3F, upper chart; control, 12.44±2.81 µm; *Myo10^−/−^*, 12.12±2.66 µm) and width (Fig. 3F, lower chart; control, 16.04±0.98 µm; *Myo10^−/−^*, 16.24±1.59 µm). Consistent with our observations on spindle assembly (Fig. 2C,D), bounding box values were not significantly different between the two conditions. Our results show that MYO10 does not transmit forces from the actin cage to the meiotic spindle by crosslinking F-actin and spindle microtubules.

### Mice lacking MYO10 in their oocytes are fertile

**Fig. 4.**
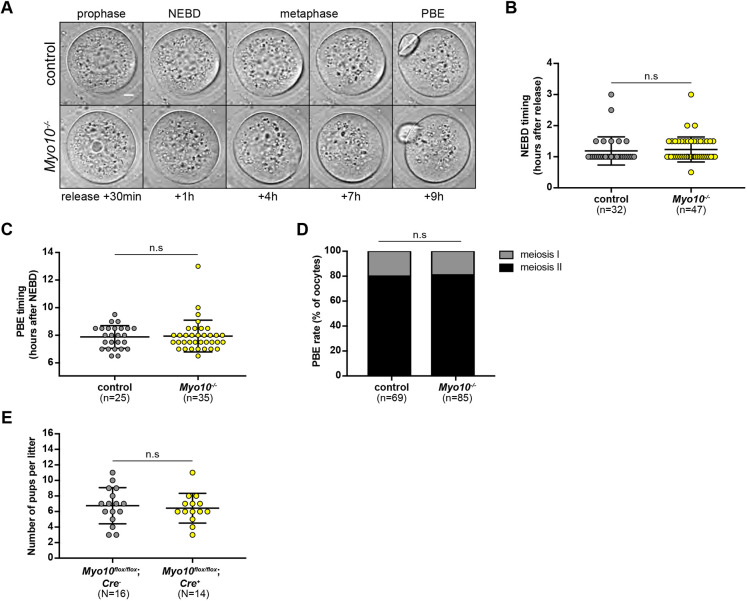
**MYO10 is dispensable for oocyte developmental potential and female fertility.** (A) Stills from time-lapse spinning disk movies of a control (upper images) and a *Myo10^−/−^* oocyte (lower images) starting 30 min after release of the prophase block until polar body extrusion (PBE). Scale bar: 10 µm. (B) Scatter-plot of NEBD timing (hours after release) of control (gray) and *Myo10^−/−^* (yellow) oocytes. *n* is the number of oocytes analyzed. Data are from three independent experiments (mean±s.d. with individual data points plotted). The statistical significance of the differences was assessed with a two-tailed Mann-Whitney test, *P*=0.2275. (C) Scatter plot of polar body extrusion (PBE) timing (hours after NEBD) of control (gray) and *Myo10^−/−^* (yellow) oocytes. *n* is the number of oocytes analyzed. Data are from three independent experiments (mean±s.d. with individual data points plotted). The statistical significance of the differences was assessed with a two-tailed Mann–Whitney test, *P*=0.6907. (D) Stacked bars of the rate of polar body extrusion (PBE) as a percentage of oocytes. Gray, oocytes arrested in meiosis I that have not extruded a polar body; black, oocytes that have extruded a polar body and are arrested in meiosis II. *n* is the number of oocytes analyzed. Data are from three independent experiments. The statistical significance of the differences was assessed with a two-sided Fisher's exact test, *P*=0.8404. (E) Scatter plots of the number of pups per litter from *Myo10*^flox/flox^*; Cre^−^* female mice (control, gray) and *Myo10*^flox/flox^*; Cre^+^* female mice (carrying *Myo10^−/−^* oocytes, yellow). *N* is the number of litters analyzed, from four (*Myo10*^flox/flox^*; Cre^−^*) and five (*Myo10*^flox/flox^*; Cre^+^*) independent couplings. Data are mean±s.d. with individual data points plotted. The statistical significance of the differences was assessed with a two-tailed unpaired *t*-test, *P*=0.6848.

Even though MYO10 was dispensable for spindle positioning, we nevertheless tested whether it was required for meiotic divisions (from NEBD to meiosis II) and more broadly for oocyte developmental potential. We monitored meiotic divisions of *Myo10*^−/−^ oocytes with a focus on the timing of polar body extrusion, as MYO10 disruption has been previously reported to extend metaphase duration in *Xenopus* embryos (Woolner et al., 2008; Sandquist et al., 2016, 2018). The kinetics and completion of meiosis I were comparable with controls in *Myo10*^−/−^ oocytes (Fig. 4A-D; Movie 5). Both NEBD timing (Fig. 4A,B; control, 1.19±0.45 h; *Myo10^−/−^*, 1.23±0.40 h) and polar body extrusion timing (Fig. 4A,C; control, 7.88±0.82 h; *Myo10^−/−^*, 7.94±1.15 h) were comparable between control and *Myo10^−/−^* oocytes. No significant difference in the rate of first polar body extrusion was measured between the two conditions (Fig. 4D; control, 79.71% of meiosis II oocytes; *Myo10^−/−^*, 81.18% of meiosis II oocytes), supporting the lack of MYO10 involvement in oocyte division.

We also determined the reproductive potential of mice producing *Myo10^−/−^* oocytes by mating *Myo10*^flox/flox^*; Cre^+^* females with control males. The number of pups per litter derived from *Myo10*^flox/flox^*; Cre^+^* mice was comparable with the *Myo10*^flox/flox^*; Cre^−^-*derived litters (Fig. 4E; *Myo10*^flox/flox^*; Cre^−^*, 6.75±2.32 pups; *Myo10*^flox/flox^*; Cre^+^*, 6.43±1.91 pups), arguing that MYO10 is not essential for the developmental potential of the oocyte and for female fertility.

## DISCUSSION

Contrary to what has been described in several models of mitotic cells and in amphibian oocytes, MYO10 is dispensable for spindle assembly and positioning in murine oocytes. These discrepancies could be explained by oocyte specific features that differ from most mitotic cells. In particular, their huge volume, their lack of centrioles and their absence of external cues and cell polarity at NEBD impose alternative mechanisms for spindle morphogenesis and positioning. Importantly, microtubules are not involved in spindle off-centering and mouse oocytes lack astral microtubules present in centriole-containing mitotic cells, potentially explaining why MYO10 is not essential in this model. Indeed, MYO10 involvement in mitotic spindle positioning is likely achieved via astral microtubules, through their pulling toward retraction fibers and the regulation of their dynamics/cortical interaction in somatic cells (Kwon et al., 2015) and their asymmetric distribution at the apical cell surface in *Xenopus* embryos (Woolner and Papalopulu, 2012). Moreover, nuclear anchoring in *Xenopus* oocytes, regulated by MYO10, is a microtubule-dependent process (Weber et al., 2004). Interestingly, unlike mitotic spindles (Woolner et al., 2008; Fink et al., 2011; Woolner and Papalopulu, 2012; Kwon et al., 2015; Sandquist et al., 2016, 2018) and *Xenopus* oocytes (Weber et al., 2004), the meiosis I spindle in mouse oocytes does not undergo oscillatory movements or rotation during metaphase. Instead, the spindle migrates in a straight manner to the closest cortex along its long axis (Verlhac et al., 2000). Spindle rotation/oscillatory movements in mitotic cells (Woolner et al., 2008; Kwon et al., 2015; Sandquist et al., 2016, 2018) and *Xenopus* oocytes (Weber et al., 2004) involve MYO10. In essence, a role for MYO10 in setting up the cell division plane has so far been described mainly for the microtubule-dependent positioning of chromosome-containing structures and in spindle rotation/oscillation during metaphase, two events that do not take place in murine meiosis I oocytes. Another possibility that could explain the differences between mouse oocytes and other models is the architecture of the cytoplasmic actin-cage surrounding the spindle, which is made of linear F-actin, as opposed to the branched subcortical F-actin likely cooperating with MYO10 to position the mitotic spindles in somatic cells (Mitsushima et al., 2010; Fink et al., 2011; Kwon et al., 2015). We can thus speculate that like myosin II, the activity of which depends on the architecture of actin networks (Reymann et al., 2012), MYO10 function could differ depending on the type of F-actin meshes, preferentially crosslinking branched F-actin to microtubules in the cytoplasm.

The lack of involvement of MYO10 in spindle migration argues for another mechanism at work in transmitting forces to the spindle in murine oocytes. This mechanism could rely on another direct actin-microtubule crosslinker; MYO7a, MYO7b and MYO15a, the other mammalian myosins known to have a MyTH4-FERM domain (which could enable them to bind directly to microtubules), have not been confirmed to be expressed at the protein level in human ovaries (Human Protein Atlas database). In addition, no study to our knowledge has pointed out their prospective function in crosslinking F-actin to microtubules, nor their involvement in spindle integrity and positioning, as they are mostly studied for their functions in actin-rich protrusions (for a review, see Weck et al., 2017). Alternatively, microtubule-based motors could be good candidates for linking the actin-cage to the meiotic spindle. Two spindle and cortex-localized kinesins, NabKin and KIF14, also colocalize with actin in *Xenopus* oocytes and human somatic cells, and are able to bind F-actin in *in vitro* assays (Samwer et al., 2013). Besides motor proteins, other direct and indirect F-actin/microtubule crosslinkers have been identified as being involved in different cellular functions and could as well be at work in meiotic spindle migration (for a review, see Dogterom and Koenderink, 2019). In addition, cytoplasmic F-actin function in spindle off-centering could depend on F-actin direct binding to chromosomes. This hypothesis is supported by the presence of F-actin aggregates accumulated in chromosomal regions oriented towards the closest cortex in nocodazole-treated mouse oocytes (Azoury et al., 2008) and by the ability of F-actin to off-center the chromosomes in such a microtubule disrupted context (Verlhac et al., 2000; Leader et al., 2002; Azoury et al., 2008).

Spindle off-centering in the murine oocyte is thus an atypical process implemented to conciliate the discard of half of the oocyte DNA content with the preservation of a rich cytoplasm enabling the egg to fulfill its developmental purpose. Deciphering how this process is orchestrated and, more specifically, how forces are transmitted from the cortex to the spindle is of significant public health interest, as defects in force transmission could trigger chromosome misalignment and subsequent egg aneuploidy (Bennabi et al., 2020) – the main cause of female infertility (Gruhn et al., 2019).

## MATERIALS AND METHODS

### Mouse strains and genotyping

*Myo10^wt/flox^* mice were generated by geneOway by inserting two *loxP* sites into the C57BL/6N mice genome in intronic sequences flanking exons 23 to 25 (according to Ensembl database) of the *Myo10* endogenous locus (Fig. 1E). This line was further crossed with *FLPo* mice [B6.129S4-Gt(ROSA)26Sor^tm2(FLP*)Sor^/J] to allow excision of the Neo cassettes flanked by FRT sites (mice obtained from the Jackson Laboratory). The resulting line was crossed with *Zp3-Cre* mice available in the lab to create the final mouse strain conditionally invalidated for myosin-X in oocytes. *loxP*-insertion sites were selected to prevent expression of the MYO10 isoform 1 (F8VQB6-1, referred to as full-length MYO10, FL-myo10) and isoform 2 (F8VQB6-2, referred to as headless MYO10, Hdl-myo10, Sousa et al., 2006). To detect *Flp*-mediated excision of the Neo cassettes flanked by FRT sites, the following PCR primers were used: 5′-ACA GCC CAT ATC ACT GTC TAG AGA CCC ATT-3′, 5′-ATC GCC TTC TAT CGC CTT GAC G-3′ and 5′-GAG GAT CCA GAC TTG GAC CCG GTC-3′. To detect *Cre*-mediated excision at the *Myo10* locus, the following PCR primers were used: for the *Cre*, 5′-GCG GTC TGG CAG TAA AAA CTA TC-3′ and 5′-GTG AAA CAG CAT TGC TGT CAC TT-3′; and for the *Myo10*, 5′-ACC CCA GTA CTT GTT CAT ACA TCC TAT ATC CTA CA-3′, 5′-GAC TAC ACC ATT CTG AAT GTG CCT GAT CTC-3′ and 5′-GAG TAT CTG CCA TCT TGT CCC TAA AGG TGG-3′. Control oocytes were collected from *Myo10^wt/flox^; Cre^+^*, *Myo10^wt/flox^; Cre^−^*, *Myo10^flox/flox^; Cre^−^* and *Myo10^Δ/flox^; Cre^−^* mice, and *Myo10^−/−^* oocytes from *Myo10^flox/flox^; Cre^+^* and *Myo10^Δ/flox^; Cre^+^* mice.

### Oocyte collection, culture and cRNA microinjection

Oocytes were collected from 2- to 5-month-old female mice following ovaries shredding (Verlhac et al., 1994) in a M2+BSA medium supplemented with milirinone (1 µM, Reis et al., 2006) to maintain them in prophase I arrest. For live cell imaging, oocytes were microinjected with cRNAs using an Eppendorf Femtojet microinjector and were left expressing protein fusions in prophase I for 30-45 min (H2B-RFP and GFP-UtrCH) or 1 h (GFP-MYO10) before being transferred into a M2+BSA milrinone-free medium to trigger meiosis resumption. Oocyte culture and live cell imaging were performed under oil at 37°C.

### Plasmids and *in vitro* transcription of cRNA

The following constructs were used: pCS2+ GFP-Myosin10(MYO10) (a gift from Nicolas Plachta, University of Pennsylvania, PA, USA), pspe3-GFP-UtrCH (Azoury et al., 2008) and pRN3-Histone(H2B)-RFP (Tsurumi et al., 2004). Plasmids were linearized with proper restriction enzymes and subsequently *in vitro* transcribed using the T3 or SP6 mMESSAGE mMACHINE (Ambion, AM1348 and 1340) transcription kits. The resulting cRNAs were purified using the RNeasy Mini Kit (Qiagen, 74104) and were centrifuged for 90 min at 4°C before being microinjected into the cytoplasm of prophase I-arrested oocytes.

### RNA extraction and RT-qPCR

Total RNA was extracted from 30 freshly collected oocytes (*Myo10^+/+^* from *Myo10^floxt/flox^; Cre^−^* mice and *Myo10^−/−^* from *Myo10^flox/flox^; Cre^+^* mice) using the RNAqueous Micro Total RNA Isolation Kit (ThermoFisher, AM1931). Oocytes were washed three times in PBS, resuspended in lysis buffer, frozen in liquid nitrogen and conserved at −80°C for 1 h. After unfreezing, RNA extraction was carried out following the manufacturer's instructions. RNA was eluted twice in 10 µl elution buffer and samples were treated with DNAse I. For reverse transcription, iScript Reverse Transcription Supermix (Bio-Rad, Ref. 1708840) was used following the manufacturer's instructions. The cDNA was then used for quantitative PCR using SsoAdvanced Universal SYBR Green Supermix (Bio-Rad, Ref. 1725270) and CFX-96 (Bio-Rad) with the primer pairs listed below. Levels were calculated using the 2-DDC. Mean and s.e.m. were both normalized relative to *Myo10^+/+^. Myo10* mRNA quantity was normalized to *Gadph*. The following primers were used: *Myo10*, 5′-GGC ACG AAA GCA ATA TAG AAA GG-3′ and 5′-CTT CTG GAA CAC GAT GGC TG-3′, *Gadph*, 5′-TGG AGA AAC CTG CCA AGT ATG-3′ and 5′-GGT CCT CAG TGT AGC CCA AG-3′.

### Immunostaining

Fully grown oocytes were freed from their zona pellucida in acid Tyrode before fixation. Granulosa-oocyte complexes (GOCs) and fully grown oocytes were fixed on gelatin and polylysine-treated coverslips.

For F-actin and α-tubulin staining, fully grown oocytes were fixed for 30 min at 37°C in PBS, EGTA (pH 7, 50 mM), HEPES (pH 7, 100 mM) and MgSO_4_ (10 mM) buffer supplemented with 2% formaldehyde (methanol free) and 0.2% Triton X-100 (Schuh and Ellenberg, 2008). Oocytes were washed and permeabilized overnight at 4°C in PBS and 0.1% Triton X-100. Blocking (30 min) and antibody incubation (primary, 1 h 30 min; secondary, 1 h) were carried out the next day at room temperature in PBS, 0.1% Triton X-100 and 3% BSA. Washes between and after the primary and secondary antibody incubations were carried out in PBS and 0.1% Tween 20. α-Tubulin staining is not shown in Fig. 2E.

For MYO10 and α-tubulin staining, fully grown oocytes were fixed for 30 min at 37°C in PBS and 4% paraformaldehyde, and washed in PBS overnight at 4°C. Oocytes were permeabilized the next day in PBS and 0.5% Triton X-100 for 10 min, and washed subsequently in PBS and in PBS/0.1% Tween 20. Blocking (30 min) and antibody incubation (primary, 1 h 30 min; secondary, 1 h) were carried out at room temperature in PBS, 0.1% Tween 20 and 3% BSA. Washes between and after the primary and secondary antibody incubations were carried out in PBS/0.1% Tween 20.

GOCs were fixed for 30 min at 37°C in PBS, EGTA (pH 7, 50 mM), HEPES (pH 7, 100 mM) and MgSO_4_ (10 mM) buffer supplemented with 0.3% formaldehyde (methanol free) and 2% Triton X-100, and washed in PBS overnight at 4°C. GOCs were permeabilized again the next day in PBS and 0.5% Triton X-100 for 10 min, and washed subsequently in PBS and PBS/0.1% Tween 20. Blocking (30 min) and antibody incubation (primary, 1 h 30 min; secondary, 1 h) were carried out at room temperature in PBS, 0.1% Tween 20 and 3% BSA. Washes between and after the primary and secondary antibody incubations were carried out in PBS/0.1% Tween 20.

Fully grown oocytes and GOCs were mounted on 0.25 mm or 0.50 mm thick slide wells, respectively, filled with Vectashield Antifade Mounting Medium with DAPI (Vector Laboratories, H-1200). We used rabbit anti-MYO10 (Sigma-Aldrich, HPA024223; 1:200) and mouse anti-α-tubulin (αTUB, Sigma-Aldrich, T8203; 1:200) primary antibodies. We used Cy3-conjugated anti-rabbit (Jackson ImmunoResearch, 11-165-152; 1:150), Cy3-conjugated anti-mouse (Jackson ImmunoResearch, 715-165-151; 1:150) and Alexa Fluor 488-conjugated anti-mouse (Invitrogen, A21121; 1:150) secondary antibodies. To label F-actin, fully grown oocytes and GOCs (not shown in Fig. 1G for GOCs) were incubated with Alexa Fluor 488-conjugated phalloidin (Invitrogen, A12379; 10 U/ml) for 1 h concomitantly with secondary antibody incubation.

### Fixed and live imaging

Spinning disk images and movies were acquired using a Plan-APO 40×/1.25 NA objective on a Leica DMI6000B microscope enclosed in a thermostatic chamber (Life Imaging Service) equipped with a Retiga 3 CCD camera (QImaging) coupled to a Sutter filter wheel (Roper Scientific) and a Yokogawa CSU-X1-M1 spinning disk. Metamorph software (Universal Imaging) was used to collect data.

### Image analysis

MYO10 spindle enrichment was quantified by measuring the ratio of the spindle and cytoplasmic MYO10 fluorescence intensities in fixed oocytes labeled with MYO10 and α-tubulin at NEBD+6 h, and in live oocytes expressing GFP-MYO10 and incubated with SiR-tubulin (from prophase I, SiR-tub, Spirochrome, SC006; 100 nM) at NEBD+6 h. Background subtraction and intensity measurements were made using Metamorph software. Spindle and cytoplasmic integrated fluorescence intensities were measured randomly four times in each compartment (spindle and cytoplasm) per oocyte using the same circle ROI of 17 μm^2^. Measurements were taken on one focal plane containing the spindle.

MYO10 cytoplasmic intensity was measured on fixed oocytes labeled with MYO10 at NEBD+6 h. F-actin cytoplasmic intensity was measured on fixed oocytes labeled with phalloidin at NEBD+6 h 30 min. Background subtraction and intensity measurements were carried out using Metamorph software. The integrated intensity was measured on the equatorial plane of the oocytes in the same ellipsoidal ROI encompassing the oocyte cytoplasm (MYO10 ellipse=3776.29 µm^2^, F-actin ellipse=2869.04 µm^2^). Intensity values were then normalized to the highest control value.

Spindle inter-polar distance, spindle width and spindle-cortex distance in meiosis I were measured manually using Metamorph software 30 min before polar body extrusion on live oocytes incubated with SiR-tub (100 nM). Only spindles parallel to the observation plane were measured. Spindle position in meiosis II was assessed visually 7 h after polar body extrusion on live oocytes incubated with SiR-tub (100 nM).

Cortical thickness (including subcortical F-actin) was measured on live oocytes expressing GFP-UtrCH at NEBD+7 h. The cortical thickness value is the mean of four measurements taken manually and randomly per oocyte on the oocyte equatorial plane (Chaigne et al., 2013; Bennabi et al., 2020) using Metamorph software.

Chromosome alignment was assessed on live oocytes expressing H2B-RFP by manually framing the chromosomes in a bounding box (Bennabi et al., 2020). The height and width of the bounding boxes were measured 30 min before anaphase using Metamorph software only on spindles parallel to the observation plane.

### Statistical analysis

Statistical analysis was carried out using GraphPad Prism software version 7.0a. For two-group comparison, the normality of each group distribution was tested with a D'Agostino and Pearson normality test. The group means were subsequently compared either with a parametric two-tailed unpaired *t*-test (with Welch's correction in the case of not equal variances for MYO10 cytoplasmic integrated intensity) or with a non-parametric two-tailed Mann-Whitney test. For the rate of polar body extrusion, the repartition of each condition was tested for statistical significance with a two-sided Fisher's exact test. Each test was carried out with a confidence interval of 95%. Error bars represent either standard deviation or standard error of mean for relative *Myo10* mRNA quantity. n.s., non-significant (*P*>0.05; ****P<0.0001).

### Ethical statement

All experimental procedures used for the project have been approved by the ministry of agriculture to be conducted in our CIRB animal facility (authorization number 75-1170). The use of all the genetically modified organisms described in this project has been granted by the DGRI (Direction Générale de la Recherche et de l'Innovation: Agrément OGM; DUO-5291).

## Supplementary Material

Reviewer comments
