# Supplementary material for: Myosin-X is dispensable for spindle morphogenesis and positioning in the mouse oocyte
Source: Development. 2021 Apr 15;148(7):dev199364. doi: 10.1242/dev.199364 (PMC8077531; doi:10.1242/dev.199364)
Supplement: Reviewer comments [file develop-148-199364_review_history.pdf]

Myosin-X is dispensable for spindle morphogenesis and positioning in the mouse oocyte

Flora Crozet, Christelle Da Silva, Marie-Hélène Verlhac and Marie-Emilie Terret
DOI: 10.1242/dev.199364

Editor: Haruhiko Koseki

Review timeline

Original submission:	14 December 2020
Editorial decision:	21 January 2021
First revision received:	11 February 2021
Editorial decision:	1 March 2021
Second revision received:	1 March 2021
Accepted:	2 March 2021

Original submission

First decision letter

MS ID#: DEVELOP/2020/199364

MS TITLE: Myosin-X is dispensable for spindle morphogenesis and positioning in mouse oocyte

AUTHORS: Flora Crozet, Christelle Da Silva, Marie-Helene Verlhac, and Marie-Emilie Terret

I have now received all the referees' reports on the above manuscript, and have reached a decision. The referees' comments are appended below, or you can access them online: please go to BenchPress and click on the 'Manuscripts with Decisions' queue in the Author Area.

As you will see, the referees express considerable interest in your work, but have some significant criticisms and recommend a substantial revision of your manuscript before we can consider publication. If you are able to revise the manuscript along the lines suggested, which may involve further experiments, I will be happy receive a revised version of the manuscript. Your revised paper will be re-reviewed by one or more of the original referees, and acceptance of your manuscript will depend on your addressing satisfactorily the reviewers' major concerns. Please also note that Development will normally permit only one round of major revision.

We are aware that you may be experiencing disruption to the normal running of your lab that make experimental revisions challenging. If it would be helpful, we encourage you to contact us to discuss your revision in greater detail. Please send us a point-by-point response indicating where you are able to address concerns raised (either experimentally or by changes to the text) and where you will not be able to do so within the normal timeframe of a revision. We will then provide further guidance. Please also note that we are happy to extend revision timeframes as necessary.

Please attend to all of the reviewers' comments and ensure that you clearly highlight all changes made in the revised manuscript. Please avoid using 'Tracked changes' in Word files as these are lost in PDF conversion. I should be grateful if you would also provide a point-by-point response detailing how you have dealt with the points raised by the reviewers in the 'Response to Reviewers' box. If you do not agree with any of their criticisms or suggestions please explain clearly why this is so.

© 2021. Published by The Company of Biologists under the terms of the Creative Commons Attribution License (https://creativecommons.org/licenses/by/4.0/).

Reviewer 1

Advance summary and potential significance to field

In the present study, authors study the function of myosin-X (MYO10) in mouse oocytes. The manuscript starts with the investigation of MYO10 localization, which showed that MYO10 was not localized on the spindle but at oocyte cytoplasm. Then authors investigate the depletion of MYO10 effect on spindle formation and migration using Cre-loxP system. They find that MYO10 depletion oocyte showed significant decrease of cytoplasmic MYO10 level, however, no effect on spindle formation and migration. The authors also find MYO10 KO oocyte had developmental potential and female fertility.

Comments for the author

While the findings presented in this manuscript are in part important for scientific community and the authors argumentation is partially conclusive, I would not recommend a publication of this manuscript in its current form, due to major concerns:

The major concern with this manuscript is that MYO10 depletion oocytes showed no phenotype. So, current form of this manuscript made not so much advance for the field. First, I recommend checking the protein level in Myo10-/- oocytes by western blotting. Because the oocyte is specific cell, sometime protein was stably stocked in cytoplasm. Also, I recommend confirming by another methods (ex. siRNA KD system). Another possibility was ZP3-Cre was not working, because some oocyte proteins were already stocked in cell before ZP3 expression. In this case, please try to use Gdf9-Cre.

Reviewer 2

Advance summary and potential significance to field

Crozet and coworkers characterize a Myosin-X conditional knock out mouse focusing on potential effects on meiotic divisions of oocytes. Despite using advanced and sensitive live cell imaging assays -- somewhat surprisingly -- the authors were unable to see any effect on either spindle morphology and movement. A phenotype would have been expected as it has been shown earlier that Myosin-X plays a major role in anchoring the meiotic spindle to the cortex in Xenopus oocytes, and later it has been demonstrated that Myosin-X has a related function in somatic cells, which is assumed to be conserved across vertebrates.

The data shown is of very high quality; figures include careful quantification of effects, appropriate controls and statistical analyses. The data shown fully support the conclusions. The text is clearly written and easy to follow, and therefore is in principle suitable for publication in Development.

The results are significant, as it is largely unexpected that Myosin-X has no effect in mouse meiosis. In particular, it is now clear that in mouse meiosis actin and actin-microtubule interactions play important roles. Myosin-X would have been the perfect candidate to coordinate these functions. Thus, this study raises the question what regulators other than Myosin-X may be involved then?

Comments for the author

The main issue of the manuscript is the general problem of negative results. Putting aside the natural human bias towards wanting to see something positive, it is for a fact that a negative result is much harder to prove than a positive one. For example, any assay has a detection threshold and in such cases the possibility always remains that the effect just falls below this threshold. For this reason, in its current form I would not oppose the publication of the manuscript, but I am also not considering it particularly strong candidate.

What would make a major difference, if the authors could compare the lack of phenotype of Myosin-X KO in oocytes to another cellular context, in which Myosin-X does have a role. Is Myosin-X required during early embryonic divisions? Or could some of the assays be carried out in mouse

© 2021. Published by The Company of Biologists under the terms of the Creative Commons Attribution License (https://creativecommons.org/licenses/by/4.0/).

fibroblasts to demonstrate the phenotype of Myosin-X KO? I am aware that carrying out these experiments is a very significant effort, but on the other hand, this would very significantly increase the impact of the paper.

Reviewer 3

Advance summary and potential significance to field

An key event of oogenesis is the execution of asymmetric cell divisions during meiosis-I and II, that lead to the formation of small polar bodies and a large egg, which contains the majority of the ooplasm. Much work by these authors and others over the past years have defined spindle migration in the oocyte as crucial – it places the spindle next to the cortex to enable the cell division to be asymmetric. This spindle migration and positioning is actin dependent. Hwever, how this works remains incompletely understood, and a high profile question. In somatic cells, Myosin 10 is often involved in actin-dependent spindle movements and positioning. This paper is straightforward and easy to read, and simply sets out to determine whether myosin-10 plays such a role in mouse oocytes. To do this the authors made an oocyte-specific Myo10 knockout using cre-lox, and examined many parameters of spindle function and migration. In essence they find that myoson-10 knockout does little to the oocyte ion this regard – cell divisions still look healthy, and are still asymmetric.

Comments for the author

The strength of the paper is the very clear genetic approach that had been taken and the clarity of the results. Albeit a 'negative result' the possibility of myo10 being involved in spindle migration was reasonable, and ruling it out so clearly is an important contribution to the field.

Specific comments
1. Is myo10 necessary for the continued anchoring of the spindle in the cortex in MetII, and thus the asymmetric nature of anaphase-II? This should be easy to add (if the authors do not already know), and would seem to complete the story.
2. Why do the authors think they have a different localisation to the So 2019 paper in Science? This is important, and should be discussed directly in the paper 3. I am not sure of the relevance of the zoomed images in Fig1 – is it being suggested that myo10 is in vesicles? Trans-zonal projections?
4. Without taking away from the importance of the paper, I actually think the rhetoric in the opening sentence of the abstract it a little too much – if a 2 cell blastomere can make a live mouse, is spindle migration really 'essential'?

First revision

Author response to reviewers' comments

We appreciate the careful review given to our manuscript " Myosin-X is dispensable for spindle morphogenesis and positioning in the mouse oocyte ". We are pleased that the reviewers were positive about the significance and novelty of our findings for the scientific community and we have sought to address all their concerns as follows. We believe that our revised manuscript is now strengthened. Please note that all changes made to the manuscript are written in red.

Reviewer 1:

- Advance Summary and Potential Significance to Field:

In the present study, authors study the function of myosin-X (MYO10) in mouse oocytes. The manuscript starts with the investigation of MYO10 localization, which showed that MYO10 was not localized on the spindle but at oocyte cytoplasm. Then, authors investigate the depletion of MYO10 effect on spindle formation and migration using Cre-loxP system. They find that MYO10 depletion

© 2021. Published by The Company of Biologists under the terms of the Creative Commons Attribution License (https://creativecommons.org/licenses/by/4.0/).

oocyte showed significant decrease of cytoplasmic MYO10 level, however, no effect on spindle formation and migration. The authors also find MYO10 KO oocyte had developmental potential and female fertility.

- Comments for the Author:

While the findings presented in this manuscript are in part important for scientific community and the authors argumentation is partially conclusive, I would not recommend a publication of this manuscript in its current form, due to major concerns: The major concern with this manuscript is that MYO10 depletion oocytes showed no phenotype. So, current form of this manuscript made not so much advance for the field. First, I recommend checking the protein level in Myo10-/- oocytes by western blotting. Because the oocyte is specific cell, sometime protein was stably stocked in cytoplasm. Also, I recommend confirming by another methods (ex. siRNA KD system). Another possibility was ZP3-Cre was not working, because some oocyte proteins were already stocked in cell before ZP3 expression. In this case, please try to use Gdf9-Cre.

We thank Reviewer #1 for these encouraging comments and for pointing out a part of our strategy that was not clear enough.

We used the ZP3-Cre strategy to conditionally invalidate Myosin-X in the oocyte because it is only active from the primary follicles, which allows to assess its function in fully-grown oocytes. Gdf9-Cre is active much earlier, in the primordial follicles, and we wanted to avoid an early depletion effect of Myosin-X because it is known that oocytes play a role in follicle activation and in the initial recruitment of follicles at this stage (for review, see Sun, Q.Y. et al Biol Reprod 2008). In addition, the use of siRNA or KD methods to confirm our results does not seem appropriate because first these methods can have off-target effects and second, they cannot provide a more efficient down-regulation of endogenous Myosin-X, hence we prefer a cleaner and more powerful genetic approach.

Regarding the efficiency of Myosin-X depletion, we show by RT-qPCR that the mRNA has disappeared (Figure 1F, note the very small error bar showing the homogeneity between

MYO10 KO oocytes), and by immunofluorescence that the protein is no longer present in fully- grown oocytes (Figure 1G-H, note the very small error bar also showing the homogeneity between MYO10 KO oocytes), both showing that the ZP3-Cre system works. We do not see the need to perform a western blot to check the level of Myosin-X protein in the oocytes, since we do it using immunofluorescence. Such a technique would require a lot of animals, which is difficult to obtain at present because of Covid (our animal facilities have significantly reduced the number of animals) and for ethical reasons (asking to reduce the unnecessary use of animals in experimentation). However, the reviewer is right, it is important to know when Myosin-X is depleted from the oocytes to exclude a possible accumulation of Myosin-X in the cytoplasm, by comparing earlier growth stages. For this purpose, we performed immunofluorescence on granulosa-oocyte complexes GOCs (new Figure 1G), comparing Control versus MYO10 KO oocytes. In Control oocytes, Myosin-X is present in the oocyte and in the surrounding follicular cells. In MYO10 KO oocytes, Myosin10 is depleted from the oocyte, but still present in the surrounding follicular cells (which also validates further our detection tool). This shows that the ZP3-Cre system is robust and that Myosin-X is depleted already at mid-growth in the oocyte, which rules out a possible stability of the protein in the cytoplasm until the fully-grown stage. We added this data to new Figure 1G and thank the reviewer for his/her suggestion.

Reviewer 2:

- Advance Summary and Potential Significance to Field:

Crozet and coworkers characterize a Myosin-X conditional knock out mouse focusing on potential effects on meiotic divisions of oocytes. Despite using advanced and sensitive live cell imaging assays -- somewhat surprisingly -- the authors were unable to see any effect on either spindle morphology and movement. A phenotype would have been expected as it has been shown earlier that Myosin-X plays a major role in anchoring the meiotic spindle to the cortex in Xenopus oocytes, and later it has been demonstrated that Myosin-X has a related function in somatic cells, which is assumed to be conserved across vertebrates. The data shown is of very high quality; figures include careful quantification of effects, appropriate controls and statistical analyses. The data shown fully support the conclusions. The text is clearly written and easy to follow, and therefore is in principle suitable for publication in Development. The results are significant, as it is largely unexpected that Myosin-

© 2021. Published by The Company of Biologists under the terms of the Creative Commons Attribution License (https://creativecommons.org/licenses/by/4.0/).

X has no effect in mouse meiosis. In particular, it is now clear that in mouse meiosis actin and actin-microtubule interactions play important roles. Myosin-X would have been the perfect candidate to coordinate these functions. Thus, this study raises the question what regulators other than Myosin-X may be involved then?

- Comments for the Author:

The main issue of the manuscript is the general problem of negative results. Putting aside the natural human bias towards wanting to see something positive, it is for a fact that a negative result is much harder to prove than a positive one. For example, any assay has a detection threshold and in such cases the possibility always remains that the effect just falls below this threshold. For this reason, in its current form I would not oppose the publication of the manuscript, but I am also not considering it particularly strong candidate. What would make a major difference, if the authors could compare the lack of phenotype of Myosin-X KO in oocytes to another cellular context, in which Myosin-X does have a role. Is Myosin-X required during early embryonic divisions? Or could some of the assays be carried out in mouse fibroblasts to demonstrate the phenotype of Myosin-X KO? I am aware that carrying out these experiments is a very significant effort, but on the other hand, this would very significantly increase the impact of the paper.

We thank Reviewer # 2 for his/her thoughtful comments. We agree that our results are "negative", but we do believe that they are important for the scientific community.

With respect to the detection threshold, we have added data strengthening our detection tool and showing that our ZP3-Cre system is robust and that Myosin-X is already depleted at mid- growth in the oocyte, which rules out a possible stability of the protein in the cytoplasm until the fully-grown stage (new Figure 1G). But the strongest evidence about the lack of role of Myosin-X in the oocyte is the absence of effect on female fertility.

Regarding the comparison between the lack of phenotype of Myosin-X KO in oocytes and another cellular context, this is why we believe that our paper is important for the scientific community, and why we discuss it at length in the discussion part. The role of Myosin-X in other cell types has been extensively studied, and adding information on its potential role in early embryonic divisions or fibroblasts would require a full knockout, outside the scope of this study which is focused on its role in the oocyte.

Reviewer 3:

- Advance Summary and Potential Significance to Field:

A key event of oogenesis is the execution of asymmetric cell divisions during meiosis-I and II, that lead to the formation of small polar bodies and a large egg, which contains the majority of the ooplasm. Much work by these authors and others over the past years have defined spindle migration in the oocyte as crucial – it places the spindle next to the cortex to enable the cell division to be asymmetric. This spindle migration and positioning is actin dependent. However, how this works remains incompletely understood, and a high profile question. In somatic cells, Myosin 10 is often involved in actin-dependent spindle movements and positioning. This paper is straightforward and easy to read, and simply sets out to determine whether myosin-10 plays such a role in mouse oocytes. To do this the authors made an oocyte-specific Myo10 knockout using cre-lox, and examined many parameters of spindle function and migration. In essence they find that myoson-10 knockout does little to the oocyte ion this regard – cell divisions still look healthy, and are still asymmetric.

- Comments for the Author:

The strength of the paper is the very clear genetic approach that had been taken, and the clarity of the results. Albeit a 'negative result' the possibility of myo10 being involved in spindle migration was reasonable, and ruling it out so clearly is an important contribution to the field.

We thank Reviewer #3 for for his/her enthusiastic comments.

Specific comments:

1. Is myo10 necessary for the continued anchoring of the spindle in the cortex in MetII, and thus the asymmetric nature of anaphase-II? This should be easy to add (if the authors do not already know), and would seem to complete the story.

© 2021. Published by The Company of Biologists under the terms of the Creative Commons Attribution License (https://creativecommons.org/licenses/by/4.0/).

Following the reviewer's question, we examined the positioning of the spindle in metaphase II-arrested oocytes. 97% of the arrested Control and KO oocytes have an off-centered spindle 7h after polar body extrusion (PBE) and even up to PBE+14h for those we followed for longer, which shows that Myosin-X is not necessary for the anchoring of the spindle in metaphase-II, in accordance with its lack of role in the oocyte and in female fertility in general. We added this data to new Figure 3C-D and thank the reviewer for his/her suggestion.

2. Why do the authors think they have a different localisation to the So 2019 paper in Science? This is important, and should be discussed directly in the paper

In the So 2019 paper, the authors used a different antibody, which is unfortunately discontinued by the company (sc-23137; Santa Cruz Biotechnology). This antibody is an affinity purified goat polyclonal antibody raised against a peptide mapping near the N-terminus of Myosin-X of human origin (https://datasheets.scbt.com/sc-23137.pdf). Since the peptide sequence is not available, we can only speculate about its precise localization within the protein sequence, but the N-terminus part of Myosin-X corresponds to its motor domain. The antibody we used is an affinity purified rabbit polyclonal antibody raised against a 152 amino-acid peptide mapping amino-acids 900 to 1051 of Myosin-X of mouse origin, covering a part of the coiled-coiled and PEST domains (Sigma-Aldrich, Ref. HPA024223). One possibility could be that the motor domain of Myosin-X has a certain degree of homology with other myosins (in particular Myosin VIIa and b), whereas the coiled-coiled and PEST domains are found only in Myosin- X.

Another difference is the immunostaining protocol used in these studies. In the So 2019 paper, oocytes are fixed in 100 mM HEPES, 50 mM EGTA, 10 mM $MgSO_4$, 2% methanol-free formaldehyde and 0.5% triton X-100 at 37°C for 15 - 60 min. They are extracted in PBS 0.5% triton X-100 (PBT) overnight at 4°C and blocked in PBT with 5% BSA (PBT-BSA) overnight at 4°C. All antibody incubations are performed in PBT-BSA at 10 µg/ml overnight at 4°C (for primary antibodies) or at 20 µg/ml for 1 h at room temperature (for secondary antibodies). This type of protocol is designed to preserve the cytoplasmic actin meshwork, but does not necessarily preserve the microtubules well. For this reason, we used a different protocol to stain microtubules and Myosin-X. We fixed oocytes for 30 min at 37°C in PBS, 4% Paraformaldehyde and washed in PBS where they stayed overnight at 4°C. Oocytes were then permeabilized the next day in PBS, 0.5% Triton X-100 for 10 min and washed subsequently in PBS and PBS, 0.1% TWEEN 20. Blocking (30 min) and antibody incubations (primary: 1h30, secondary: 1 hour) were done at room temperature in PBS, 0.1% TWEEN 20, 3% BSA.

To summarize, both the differences in antibodies and protocols could explain the different localizations observed in the So paper and in our study. However, the So paper was not intended to address the role of Myosin-X in oocytes, so the specificity of their staining was not tested on oocytes depleted for Myosin-X (genetically or at least using siRNA) as we do here.

3. I am not sure of the relevance of the zoomed images in Fig1 - is it being suggested that myo10 is in vesicles? Trans-zonal projections?

The zoomed image reinforced the fact that our detection tool was robust and indeed specific to Myosin-X. It was shown by El-Hayek S et al Curr Biol 2018 that Myosin-X is localized in the Trans-zonal projections (TZPs) coming from the somatic cells surrounding the oocyte. Thus, in KO oocytes, the oocyte Myosin-X staining is lost, but the signal coming from the somatic cells in the TZPs is present. We have also added new data strengthening our detection tool and showing that in KO growing oocytes, Myosin-X is already depleted from the oocyte, but still present in the surrounding follicular cells. This shows that the ZP3-Cre system is robust and that Myosin-X is already depleted as of the mid-growth stage in the oocyte, which rules out a possible stability of the protein in the cytoplasm until the fully-grown stage. We added this data to the new Figure 1G.

4. Without taking away from the importance of the paper, I actually think the rhetoric in the opening sentence of the abstract it a little too much - if a 2 cell blastomere can make a live mouse, is spindle migration really 'essential'?

We agree that a 2-cell blastomere can make a live mouse, certainly after ZGA, and with the contribution of the paternal genome. Thus, we have replaced "essential" by "important" in the

© 2021. Published by The Company of Biologists under the terms of the Creative Commons Attribution License (https://creativecommons.org/licenses/by/4.0/).

abstract. However, Kyogoku et al Dev Cell 2017 actually fertilized halved oocytes, and showed that they have a lower developmental potential (only 30% of them reached the blastocyst stage, compared to 90% for normal-sized oocytes).

Second decision letter

MS ID#: DEVELOP/2020/199364

MS TITLE: Myosin-X is dispensable for spindle morphogenesis and positioning in the mouse oocyte

AUTHORS: Flora Crozet, Christelle Da Silva, Marie-Helene Verlhac, and Marie-Emilie Terret

I have now received all the referees reports on the above manuscript, and have reached a decision. The referees' comments are appended below, or you can access them online: please go to BenchPress and click on the 'Manuscripts with Decisions' queue in the Author Area.

The overall evaluation is positive and we would like to publish a revised manuscript in Development, provided that the referee 3's point can be appropriately incorporated. If you do not agree with any of their criticisms or suggestions explain clearly why this is so.

We are aware that you may currently be unable to access the lab to undertake experimental revisions. If it would be helpful, we encourage you to contact us to discuss your revision in greater detail. Please send us a point-by-point response indicating where you are able to address concerns raised (either experimentally or by changes to the text) and where you will not be able to do so within the normal timeframe of a revision. We will then provide further guidance. Please also note that we are happy to extend revision timeframes as necessary.

Reviewer 1

Advance summary and potential significance to field

This is a revision of a manuscript that presents a study of myosin-X (MYO10) function in mouse oocytes. Authors have clearly addressed reviewer's comment. The results are still "negative", but the results are important for scientific community. I support publication of the revised manuscript.

Comments for the author

The authors have addressed my concerns and the manuscript is now suitable for publication.

Reviewer 2

Advance summary and potential significance to field

See my previous comments on the original submission.

Comments for the author

My overall opinion remains unchanged. I find the paper clearly written, the data are very high quality and they clearly and strongly support the conclusions drawn. During the revisions a few important, additional controls have been added which further strengthened these conclusions (e.g. Fig. 1G).

© 2021. Published by The Company of Biologists under the terms of the Creative Commons Attribution License (https://creativecommons.org/licenses/by/4.0/).

Thereby, the authors show using a knock out mouse model that MyoX is dispensable for mouse oocyte meiosis. This is an unexpected finding with key importance for the community. At the same time, it is a negative result, for example, it does not provide an explanation for how the processes which normally require MyoX operate in the oocyte in a MyoX independent manner.

Nevertheless, overall I am in support of publication of the manuscript in Development.

Reviewer 3

Advance summary and potential significance to field

As previously.

Comments for the author

The authors have answered my previous questions, including adding MetII data, which is a good addition. This is a nice paper that is interesting to the field.

It is my opinion that that authors should discuss in their paper (as opposed to just to the reviewer) the difference in IF results between this paper and the So Science paper, at least a little bit.

Second revision

Author response to reviewers' comments

We appreciate the careful review given to our manuscript " Myosin-X is dispensable for spindle morphogenesis and positioning in the mouse oocyte ". We are pleased that the reviewers were satisfied with our revisions of the manuscript. Please note that all changes made to the manuscript in response to reviewer 3 are written in red.

Reviewer 1:

- Advance Summary and Potential Significance to Field: This is a revision of a manuscript that presents a study of myosin-X (MYO10) function in mouse oocytes. Authors have clearly addressed reviewer's comment. The results are still "negative", but the results are important for scientific community. I support publication of the revised manuscript.
- Comments for the Author: The authors have addressed my concerns and the manuscript is now suitable for publication.
-

Thanks a lot.

Reviewer 2:

- Advance Summary and Potential Significance to Field: See my previous comments on the original submission.
- Comments for the Author: My overall opinion remains unchanged. I find the paper clearly written, the data are very high quality and they clearly and strongly support the conclusions drawn. During the revisions a few important, additional controls have been added, which further strengthened these conclusions (e.g. Fig. 1G).

Thereby, the authors show using a knock out mouse model that MyoX is dispensable for mouse oocyte meiosis. This is an unexpected finding with key importance for the community. At the same time, it is a negative result, for example, it does not provide an explanation for how the processes which normally require MyoX operate in the oocyte in a MyoX independent manner.
Nevertheless, overall I am in support of publication of the manuscript in Development.

Thanks a lot.

© 2021. Published by The Company of Biologists under the terms of the Creative Commons Attribution License (https://creativecommons.org/licenses/by/4.0/).

Reviewer 3:

- Advance Summary and Potential Significance to Field: As previously.
- Comments for the Author: The authors have answered my previous questions, including adding MetII data, which is a good addition. This is a nice paper that is interesting to the field. It is my opinion that that authors should discuss in their paper (as opposed to just to the reviewer) the difference in IF results between this paper and the So Science paper, at least a little bit.

We added a short discussion in the text regarding this point.

Third decision letter

MS ID#: DEVELOP/2020/199364

MS TITLE: Myosin-X is dispensable for spindle morphogenesis and positioning in the mouse oocyte

AUTHORS: Flora Crozet, Christelle Da Silva, Marie-Helene Verlhac, and Marie-Emilie Terret
ARTICLE TYPE: Research Article

I am happy to tell you that your manuscript has been accepted for publication in Development, pending our standard ethics checks.

© 2021. Published by The Company of Biologists under the terms of the Creative Commons Attribution License (https://creativecommons.org/licenses/by/4.0/).
